# Automated classification in turtles genus *Malayemys* using ensemble multiview image based on improved YOLOv8 with CNN

**DOI:** 10.1038/s41598-024-76431-9

**Published:** 2024-10-23

**Authors:** Wararat Songpan, Thotsapol Chaianunporn, Khemika Lomthaisong, Sarun Keithmaleesatti

**Affiliations:** 1 Department of Computer Science, College of Computing, Khon Kaen University, Khon Kaen, 40002 Thailand; 2 Department of Environmental Science, Faculty of Science, Khon Kaen University, Khon Kaen, 40002 Thailand; 3 Forensic Science Program, Faculty of Science, Khon Kaen University, Khon Kaen, 40002 Thailand

**Keywords:** Automated classification, Multiview image, YOLOv8, CNN, *Malayemys*, Conservation, Turtle, Classification and taxonomy, Machine learning

## Abstract

In Thailand, two snail-eating turtle species in the genus *Malayemes* (*M. subtrijuga* and *M. macrocephala*) are protected animals in which smuggling and trading are illegal. Recently, a new species *M. khoratensis* has been reported and it has not yet been considered as protected animal species. To enforce the law, species identification of *Malayemes* is crucial. However, it is quite challenging and requires expertise. Therefore, a simple tool, such as image analysis, to differentiate these three snail-eating species would be highly useful. This study proposes a novel ensemble multiview image processing approach for the automated classification of three turtle species in the genus *Malayemys*. The original YOLOv8 architecture was improved by utilizing a convolutional neural network (CNN) to overcome the limitations of traditional identification methods. This model captures unique morphological features by analyzing *Malayemys* species images from various angles, addressing challenges such as occlusion and appearance variations. The ensemble multiview strategy significantly increases the YOLOv8 classification accuracy using a comprehensive dataset, achieving an average mean average precision (mAP) of 98% for the genus *Malayemys* compared with the nonensemble multiview and single-view strategies. The species identification accuracy of the proposed models was validated by comparing genetic methods using mitochondrial DNA with morphological characteristics. Even though the morphological characteristics of these three species are ambiguous, the mitochondrial DNA sequences are quite distinct. Therefore, this alternative tool should be used to increase confidence in field identification. In summary, the contribution of this study not only marks a significant advancement in computational biology but also supports wildlife and turtle conservation efforts by enabling rapid, accurate species identification.

## Introduction

All *Malayemys* species are native freshwater turtles that are endemic to mainland Southeast Asia. Based on morphological and molecular characteristics, including microsatellite DNA and mitochondrial DNA, it was revealed that there are three species of snail-eating turtle, (1) Malayan snail-eating turtle *M. macrocephala* distributed in northern, central, and southern Thailand, east Cambodia, and Malaysia (2) Mekong snail-eating turtle *M. subrtijuga* distributed in Cambodia, southern Vietnam, southern Laos, and the lower Mun River in northeastern Thailand and Java (Indonesia); and (3) The Khorat snail-eating turtle *M. khoratensis* distributed in northeastern Thailand and central Laos^1–5^. The morphological characteristics, such as the pattern of the first vertebral scute, parts of lower marginal scutes 8–12, the position and shape of the infraorbital stripe, the number of nasal stripes, the number of orbital rings, and the postocular stripe, have been used to identify *Malayemys* species. However, these characteristics of each *Malayemys* species are very similar and sometimes difficult to use for species identification, whereas their DNA characteristics are quite distinct.

The International Union for Conservation of Nature (IUCN) reported that *M. subtrijuga* is near threatened (NT) while *M. macrocephala* and *M. khoratensis* are the species of least concern (LC)^6^. In Thailand, only *M. subtrijuga* and *M. macrocephala* are classified as protected species by the Wild Animal Conservation and Protection Act, B.E. 2562 (2019), while *M. khoratensis* is not even though they are facing the same threats. Some local people in mainland Southeast Asia, such as Thailand, Cambodia and Laos, still consume meat and eggs of freshwater turtles. Thus, snail-eating turtles are commonly found in local fresh markets or fish markets in these countries^3–5^. In Thailand, they are commonly available for purchase at local markets primarily due to of their significance in religious practices among Thai people^3,5^. Furthermore, these turtle species are used in traditional medicine in China and Southeast Asia. The trade and consumption of freshwater turtles by humans are responsible for the decline in freshwater turtle populations and loss of biodiversity. Moreover, the turtle trade has made species identification more difficult, as the distribution of turtles is not reliable due to their transfer between locations through trade routes.

Thailand government officials and some biologists have used the morphological characteristics of snail-eating turtles to identify turtle species (as shown in Table 1) in the field. However, we found that this method relies on human judgment and may not differentiate turtle species because of potential biases toward specific species. Consequently, a multiview analysis approach has been proposed to enhance decision reliability and accuracy by integrating weighted contributions from various perspectives obtained through deep learning approaches^7–10^. Recent advancements in the field have focused on enhancing the efficiency and accuracy of deep learning models in environmental applications. Techniques such as transfer learning, where a model developed for one task is adapted for another related task, have proven effective in scenarios where data are scarce. Additionally, efforts have been directed toward developing models that are resilient to variations in image quality and environmental conditions^11,12^.

YOLOv8 Object detection^13,14^, a pivotal advancement within the domain of computer vision and deep learning, significantly enhances the image analysis capabilities of models. Recent developments in segmentation algorithms have contributed to this progress^15–17^. These models are extensively applied in critical fields such as satellite imagery^18^, agriculture^19^, facial imaging^20^, and medical imaging^21^, as well as the biological scientific domain^22,23^. In^24–29^ refers the turtle domain, However, this research has not been the turtle can object detection in single characteristic and specific sea turtle. Despite the ability of object detection techniques to provide precise details about the locations of objects and effectively manage occlusion, achieving real-time detection with both high speed and reliable accuracy remains a substantial challenge.

Therefore, this study focuses on the development of a predictive model for identifying turtle species in the genus *Malayemys* from images and explores the effectiveness of various perspectives in species identification when expert analysis is unavailable^11,30^. The study employs a range of deep learning techniques for automated feature extraction and classification, aiming to identify which perspectives or combinations thereof are most informative for species recognition. We anticipate that certain perspectives will consistently yield high accuracy across diverse algorithms. This expectation extends to the use of YOLOv8 improved by CNNs trained specifically using images from the genus *Malayemys*. We predict improvements in accuracy. However, we also hypothesize that the comparative significance of different perspectives will remain constant across methods, assuming that no overfitting occurs with highly specialized CNNs. This study makes four key contributions to the literature:

(1) Analysis of the information from four methods for identifying *Malayemys* turtle species.

(2) Development of a combined method for accurate species identification.

(3) Evaluation and comparison of how the accuracy varies for each view with the proposed model.

(4) Automation and transfer of the best model to a web application.

The organization of the paper is as follows: “Materials and methods” provides an overview of the material and methods, and “Proposed model” outlines the proposed model framework. “Experimental results” presents the experimental results for evaluating the models and a discussion of their implementation. “Conclusions” presents the conclusions of this study. We believe that artificial intelligence (AI) is a tool for freshwater turtle conservation because it is noninvasive, fast and easy to use in field identification.

## Materials and methods

### Ethics

The Department of Fisheries, Ministry of Agriculture and Cooperatives, Thailand, granted permission for this study (DF. 1/2566). The protocol was approved by the Committee on the Ethics of Animal Experiments (permit number: IACUC-KKU-84/66) of Khon Kaen University. All animal handling was in accordance with accepted wildlife husbandry standards of American Society of Ichthyologists and Herpetologists (2004).

## Study area and sampling

**Fig. 1:**
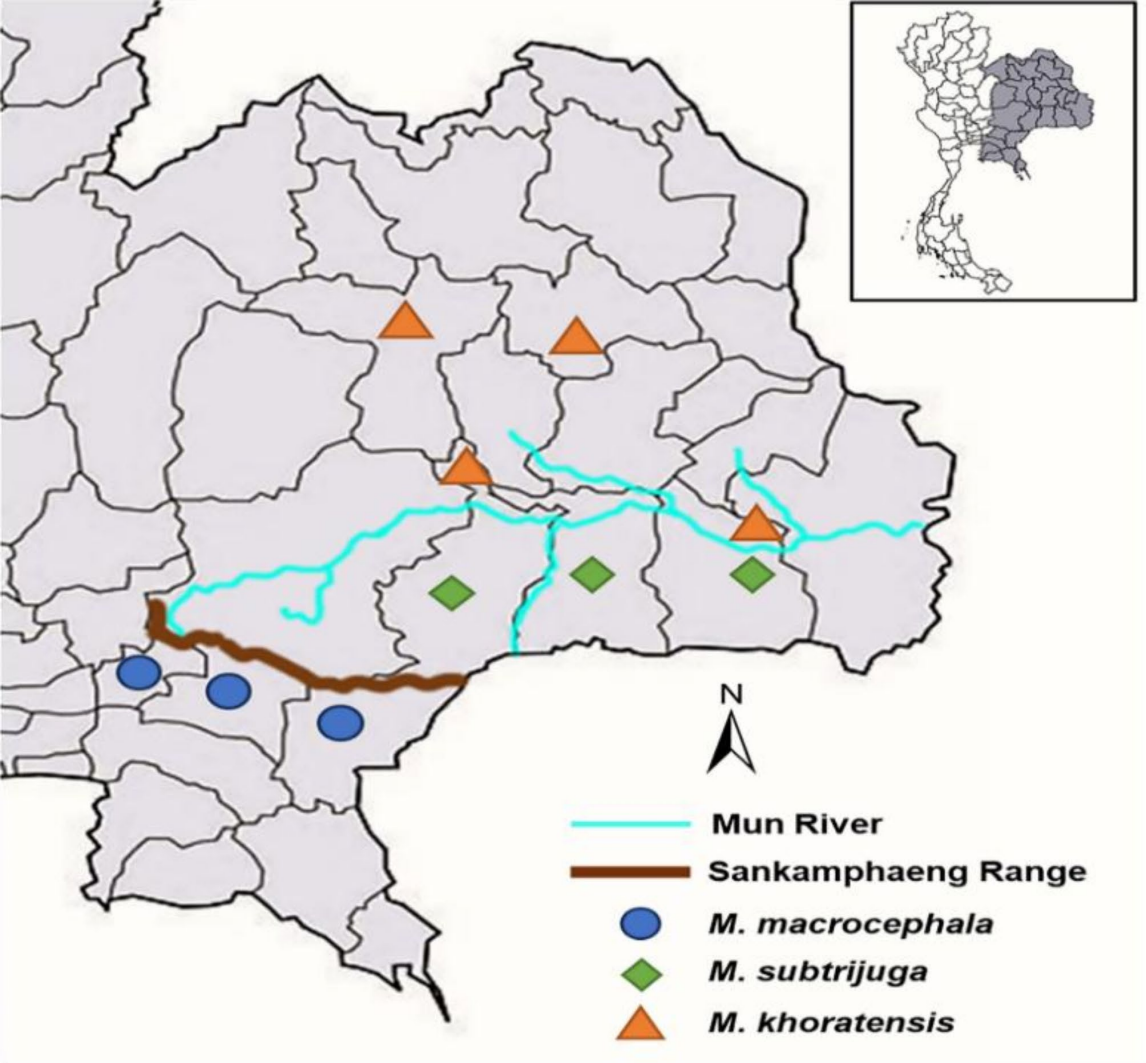
The study area map shows Thailand and the sampling locations for the *Malayemys* turtles. The black lines represent provincial borders, the blue line shows the Mun River, which separates the distributions of *M. subtrijuga* and *M. khoratensis*, and the brown line represents the Sankamphaeng Range, which separates the distributions of *M. subtrijuga* and *M. macrocephala*. The symbols indicate turtle species: blue circles for *M. macrocephala*, green diamonds for *M. subtrijuga*, and orange triangles for *M. khoratensis*.

Three snail-eating turtle species (genus *Malayemys*) were captured via hand or fish traps by local people from natural wetlands, including rice fields, canals, and ponds, in the rainy season. Six to ten images of each turtle were captured, including the front of face (nasal stripe, infraorbital stripe, and supraorbital stripe), side of face (postorbital stripe and loreal seam), carapace and plastron. All the turtles were photographed and then released into their natural habitat close to where they were captured. *M. macrocephala* was captured from eastern Thailand, including Prachin Buri Province, Sa Kaeo Province and Nakhon Nayok Province. *M. subtrijuga* was captured in northeastern Thailand, including Nakhon Ratchasima Province, Buri Ram Province, Surin Province, and Si Sa Ket Province. *M. khoratensis* was captured in northeastern Thailand, including Khon Kaen Province, Kalasin Province and Roi Et Province (Fig. 1).

**Table 1:**
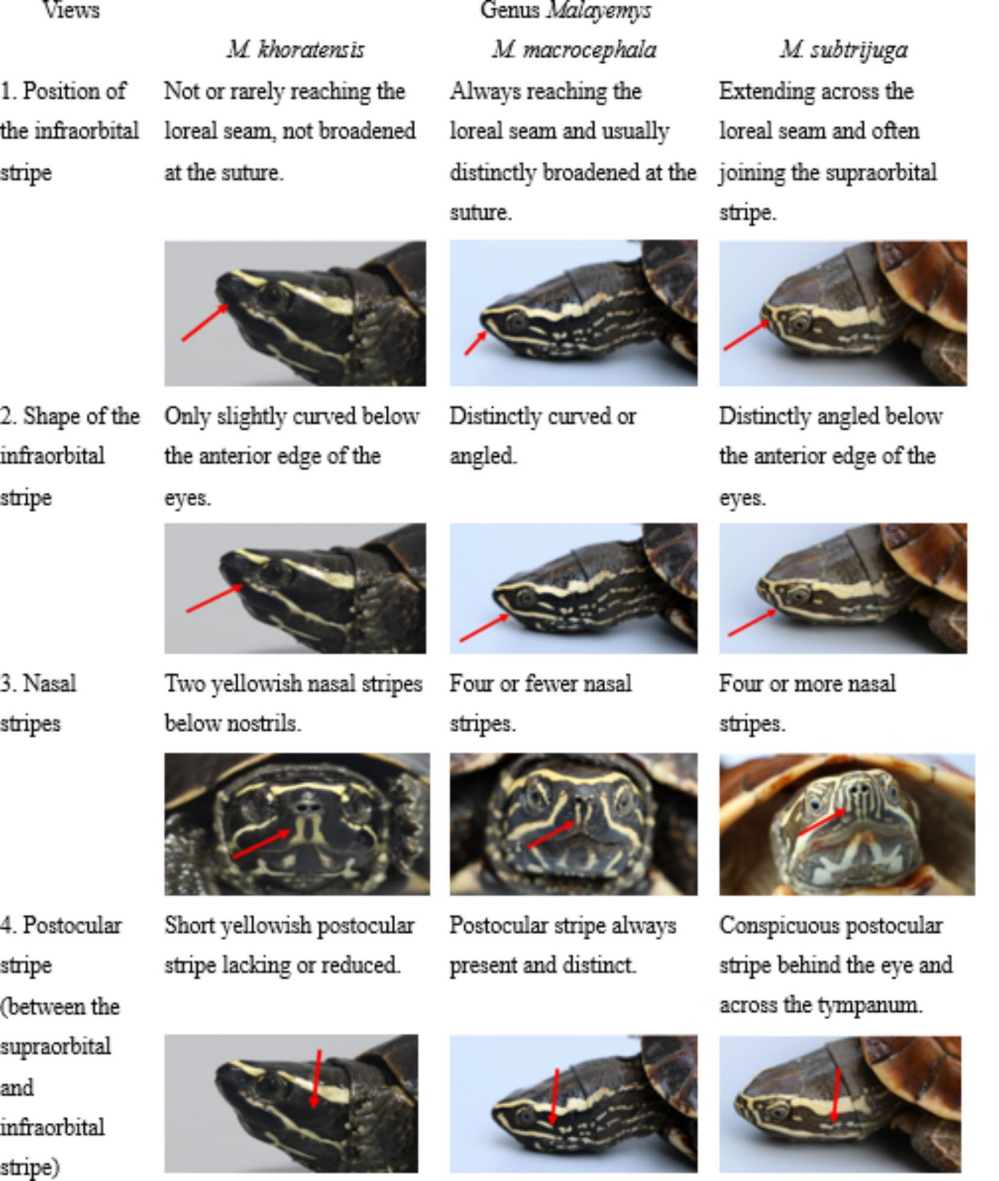
Single-view strategy analysis for the classification and identification of the genus *Malayemys*.

As Table 1, experts were able to identify the 3 species in the genus *Malayemys*, namely, *M. khoratensis*,* M. macrocephala* and *M. subtrijuga*, by utilizing four possible singular views. First, the position of the infraorbital stripe is a point of the difference line of species. For example, *M. khoratensis* has not or rarely reached the loreal seam and has not broadened at the suture, whereas *M. macrocephala* always has a line pattern reaching the loreal seam and is usually distinctly broad at the suture. *M. subtrijuga* has been characterized as extending across the loreal seam and often joining the supraorbital stripe. Second, the shape of the infraorbital stripe is slightly curved below anterior edge of the eyes of *M. khoratensis*, whereas that of *M. macrocephala* and *M. subtrijuga* is distinctly curved or angled but slightly different in angled pattern. Third, nasal strips are a strange point of view described by the number of yellowish nasal stripes. For example, *M. khoratensis* has two yellowish nasal stripes, whereas *M*. *macrocephala* and *M. subtrijuga* have four or more nasal stripes below the nostrils, respectively. Finally, the postocular stripe (between the supraorbital and infraorbital stripes) is used as a point of comparison. However, the analysis from the single-view perspective mentioned above may complicate identification.

The main goal of this study is to propose a multiview strategy that helps in making decisions on the classification and identification of the characteristics of the genus *Malayemys* from many expert perspectives to solve the problem in the case of a single view with some characteristics. The ensemble method leverages weak learners to construct a more robust learner, thereby reducing errors during training^31–33^. This approach encompasses a variety of strategies. In the bagging technique, random subsets of data of identical size are selected independently^34,35^. These subsets are then trained via different classifiers, i.e., the first subset with the first classifier, the second subset with the second classifier, and so on up to the Nth subset with the Nth classifier. The outcomes from these base classifiers are then aggregated via majority voting to predict the result. In contrast, the boosting strategy begins with the initial dataset being processed by a weak classifier. Data points that are incorrectly classified are then assigned increased weights, creating a weighted dataset. This adjustment aims to reduce bias during the training of subsequent weak classifiers. This process of weighing and reclassifying misclassified data is repeated with successive weak classifiers until the most effective weak classifier is determined.

## Proposed model

In the proposed framework, the ensemble Multiview image strategy is meticulously designed to extend the capabilities of YOLOv8 through the integration of a convolutional neural network (CNN)^12^. This extension aims to address the intrinsic limitations associated with single-view image processing in object detection applications. By leveraging multiple views of the same object, the system significantly improves the accuracy and robustness of the detection process. The CNN component is specifically designed to extract and synthesize features from diverse perspectives, enabling the YOLOv8 model to achieve a more comprehensive understanding of the object’s characteristics. This strategic integration not only enhances the detection precision but also minimizes the chances of misclassification, thereby making the system highly effective in complex visual environments where traditional single-view approaches may struggle. In addition, this framework improves the YOLOv8 model by integrating a convolutional neural network to process multiple views of an object, improving the detection accuracy and reducing misclassification in complex environments.

**Fig. 2:**
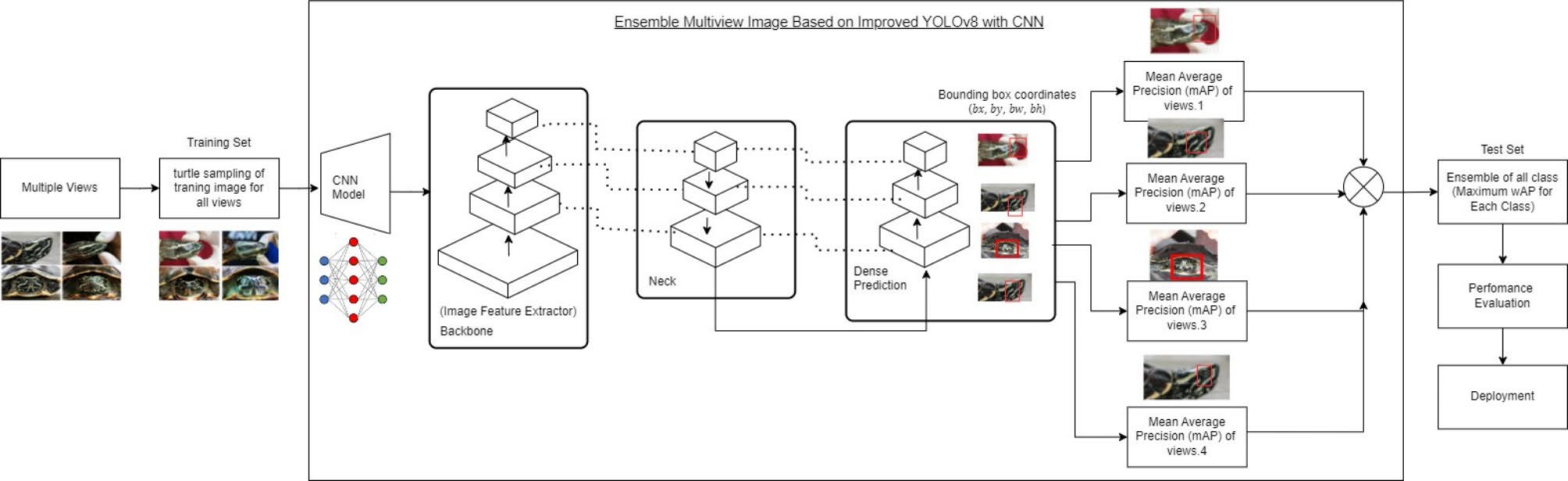
Framework of the ensemble multiview image based on improved YOLOv8 with CNN.

Figure 2 presents an ensemble-based multiview image processing approach utilizing an enhanced YOLOv8 model integrated with a convolutional neural network (CNN)^13^. The system processes multiple views of turtle images, capturing different angles to create a diverse training dataset. These images are sampled to ensure comprehensive coverage of each object’s appearance under varying conditions, such as lighting and orientation. The CNN, acting as a feature extractor, generates detailed feature maps that encapsulate essential visual elements such as edges, textures, and shapes. These feature maps are refined through the neck layer, which improves multiscale detection capabilities, enhancing the robustness of predictions. The dense prediction layer then generates bounding boxes, defined by center coordinates (x, y)(x, y)(x, y), width (bw)(bw)(bw), and height (bh)(bh)(bh), to localize detected objects within the images. For each view, the system computes the mean Average Precision (mAP)^27^, allowing for a detailed evaluation of detection accuracy across different perspectives. An ensemble method integrates the predictions, selecting the maximum mAP score for each class to achieve the most accurate detection results. Following this, the model undergoes a performance evaluation using a separate test set to ensure it generalizes well to unseen data, demonstrating consistency and reliability across various scenarios. Finally, the model is ready for deployment, offering a robust solution for applications such as turtle identification and general object detection. The ensemble approach, combined with multiview processing, enhances detection accuracy and robustness, making the framework highly effective for real-world applications. Each single view is trained and set to find out the mean Average Precision (mAP).

In improving of YOLOv8 object detection tasks, the mAP is a widely used metric to evaluate the performance of models when integrated with convolutional neural networks (CNNs). The mAP provides a comprehensive measure that captures both the precision and recall of the detector across various threshold levels, offering an aggregate performance score that reflects the accuracy of detecting multiple classes of objects. The maximum of weighted mean Average Precision (mAP) for an ensemble of object detection models for each view is calculated as follows:

1 $$\:Maximum\:of\:weighted\:{mAP}_{i}=\frac{1}{N}{\sum\:}_{j=1}^{N}{weighted\:mAP}_{j}$$

where is the number of classes for each view i.

The described process facilitates progression toward a testing data set that enables a comparison of single and multiview images. This comparison aims to identify the most effective model for subsequent applications. The performance evaluation^24–27^ for proposed model using the precision, recall and accuracy rates is computed at various confidence thresholds for each object class in Eqs. (2–4) respectively.

2 $$\:{Precision}_{ij}=\frac{{TP}_{ij}}{{TP}_{ij}+{FP}_{ij}}$$

3 $$\:{Recall}_{ij}=\frac{{TP}_{ij}}{{TP}_{ij}+{FN}_{ij}}$$

4 $$\:{Accuracy}_{ij}=\frac{{TP}_{ij}+\:{TN}_{ij}}{{TP}_{ij}+{TN}_{ij}+{FN}_{ij}+{FP}_{ij}}$$

where* i* indexes the view and* j* indexes the class.

This evaluation ensures that the model generalizes effectively to unseen data and delivers consistent results across various testing conditions. Upon successful evaluation, the model is then ready for deployment in practical applications. The ensemble approach, combined with Multiview processing, enhances detection accuracy and robustness, making the framework highly effective for real-world applications.

## Results

The dataset comprises 93 images of 3 species for training and 52 images for testing. The datasets include common benchmark datasets in the field. These datasets provide a variety of image scenarios to test the robustness of the models. The models were trained via a combination of stochastic gradient descent and adaptive learning rate techniques to optimize their performance. The training process is detailed below, with a specific focus on the adjustments made to accommodate the unique features of YOLOv8 in conjunction with CNNs. The performance of the integrated models was evaluated on the basis of precision, recall, and accuracy rates, which are assigned a value scale between 0 and 1, where 1 is the highest performance for classification. YOLOv8 with a CNN architecture achieved superior performance in various metrics compared with that of other models, highlighting its effectiveness in practical implementations. The experimental results revealed that the highest classification accuracy was 0.98 for the ensemble multiview image strategy. In terms of the single-view strategies for nasal stripes, the accuracy rate with single-view 3 was 0.82, that for single-view 2 was 0.81, and that for single-view 4 was 0.81. The accuracy rate with single-view 1 was lower than that with the other views at 0.77. The *Malayemys* species include *M. khoratensis (Khorat)* and *M. subtrijuga (Sub.)* The precision, recall, and accuracy rates were the highest for the ensemble multiview strategy. For the classification of *M. macrocephala (Mac.)*, the precision, recall, and accuracy rates were 0.94, 1.00, and 0.94, respectively. For the single-view strategies, the classification of *M. khoratensis* (*Khorat*) yielded the highest values, followed by those of *M. subtrijuga* (*Sub.*) and *M. khoratensis* (*Khorat*), respectively, as shown in Table 2.

**Table 2:** Performance of ensemble multiview strategies compared with that of single-view strategies via YOLOv8 with a convolutional neural network.

View strategy	Malayemys species									Average
	M. khoratensis (Khorat) *N = 24 Samples*			M. macrocephala (Mac.) *N = 16 Samples*			M. subtrijuga (sub.) *N = 12 Samples*			Overall
	Precision	Recall	Acc.	Precision	Recall	Acc.	Precision	Recall	Acc.	Acc.
Single View 1	0.83	0.95	0.80	0.56	0.90	0.56	0.75	0.90	0.71	0.77
Single View 2	0.91	0.95	0.88	0.65	0.90	0.58	0.83	0.90	0.76	0.81
Single View 3	0.88	1.00	0.88	0.61	0.91	0.58	0.84	0.91	0.79	0.82
Single View 4	0.91	0.95	0.88	0.65	0.90	0.58	0.83	0.90	0.76	0.81
Ensemble multiview (proposed model)	1.00	1.00	1.00	0.94	1.00	0.94	1.00	1.00	1.00	0.98

**Fig. 3:**
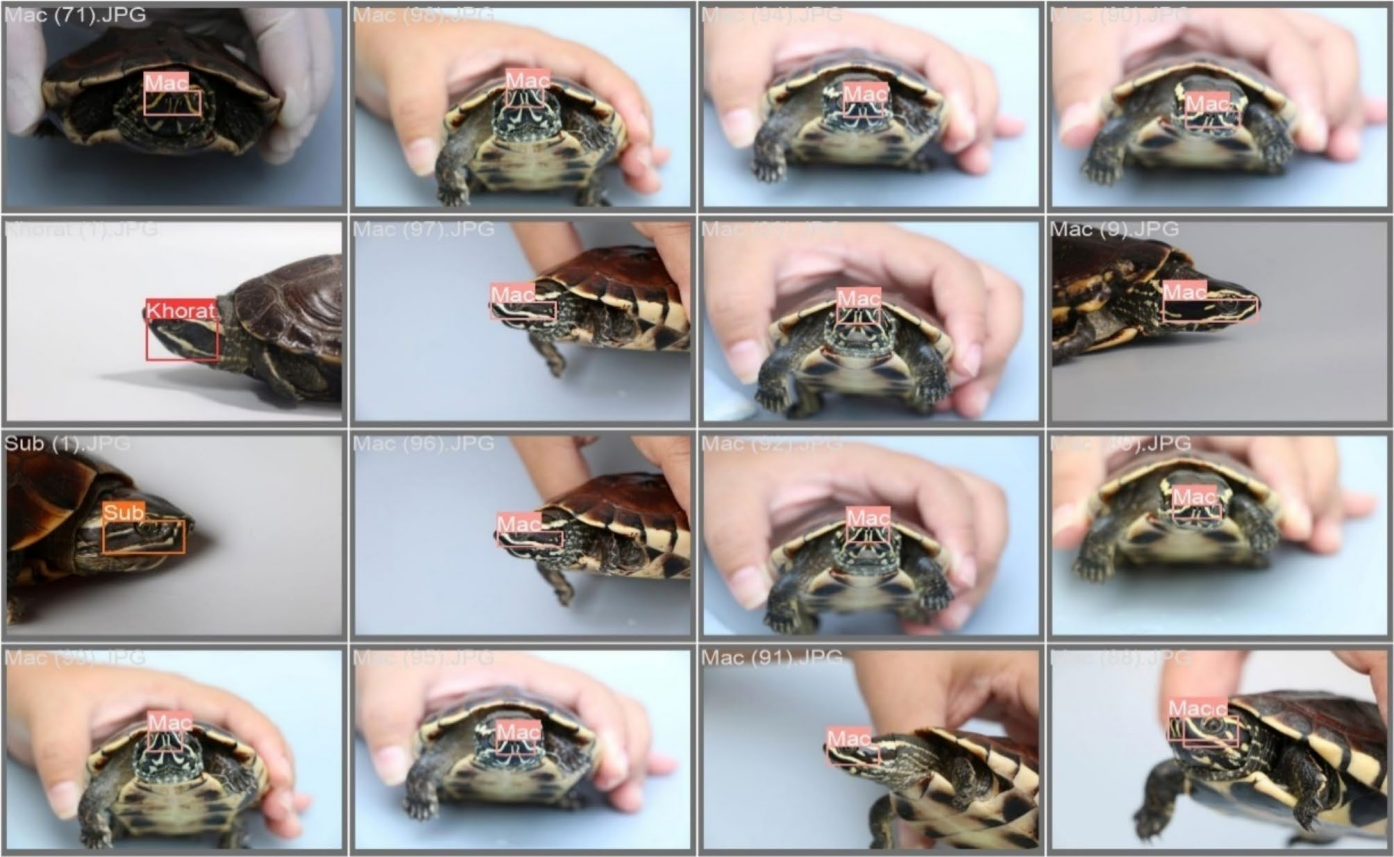
True positive classification of the genus *Malayemys*.

True positive refers to the number of images that are classified correctly in the class with the best performance, as shown in Fig. 3. The true positive classification of the genus *Malayemys* is shown in the boundary boxes. The maximum of weighted mAP of the ensemble with multiple views is calculated via Eq. (1) and represents the best point of view.

**Fig. 4:**
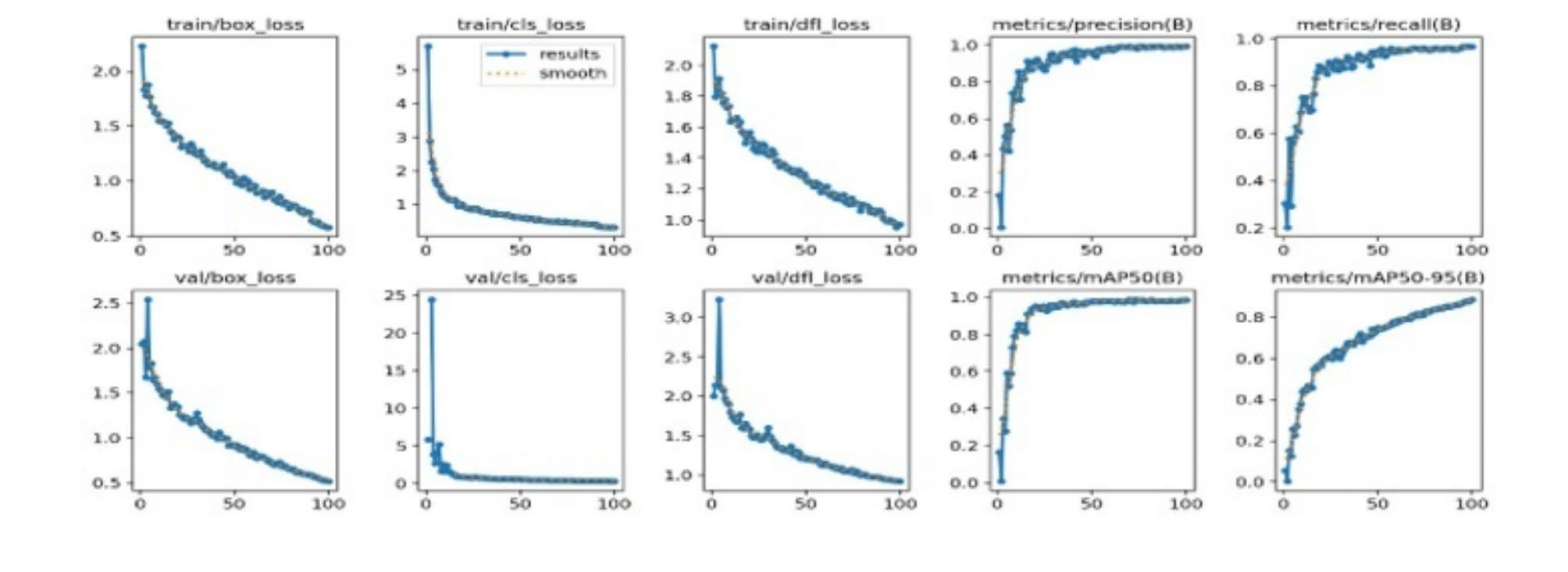
Loss function, precision and recall for the classification of the genus *Malayemys* for each epoch.

As shown in Fig. 4, the graph presents the training and validation loss metrics, as well as the precision and recall performance, for a machine learning model used to classify the genus *Malayemys* across various epochs. During training, consistent decreases were observed for both the box loss and classification loss, suggesting that the model increasingly adapts to the training data, improving its ability to localize and classify the object correctly. The displacement loss (dfl_loss) decreased observed in both the training and validation phases indicates better alignment of the predicted bounding boxes with the ground truth, and the best performance was 0.0. However, the precision and recall metrics are key indicators of the model’s accuracy and completeness, respectively, and significantly improve as training progresses. The precision steadily approaches 1.0, demonstrating that the model accurately identifies positive samples with few false positives. The recall also approaches 1.0, indicating the model’s increasing success in detecting all relevant instances within the dataset. This convergence of precision and recall, as well as the decreases in loss values, demonstrates the model’s effective learning ability and increasing robustness in the classification of the genus *Malayemys*.

**Fig. 5:**
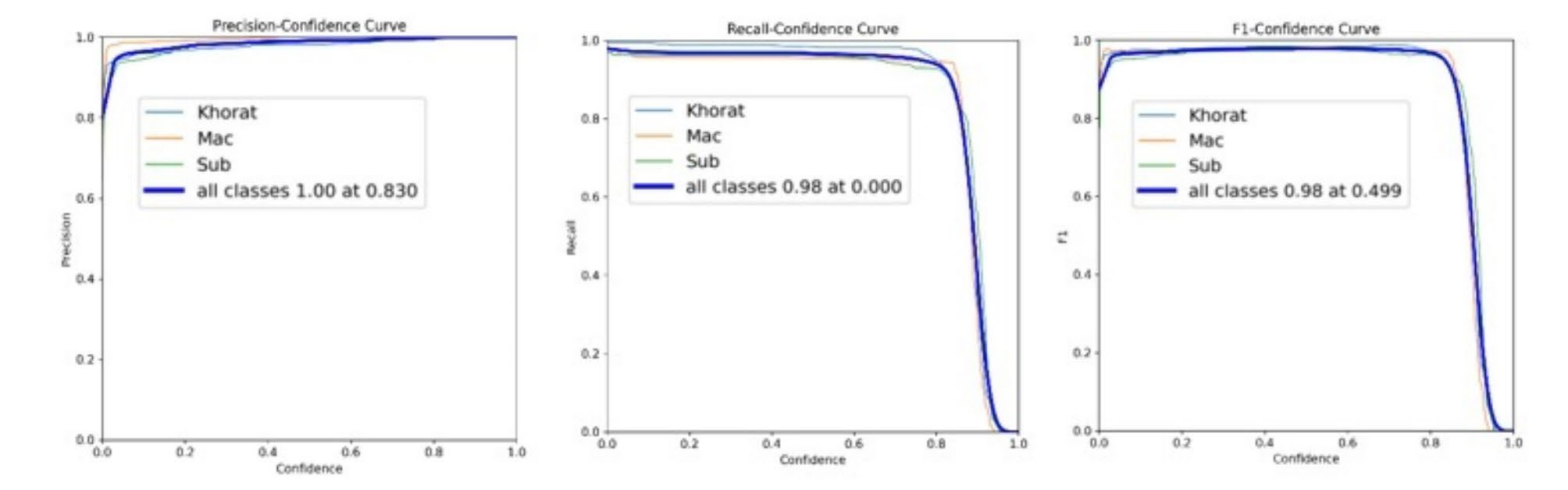
Precision, recall, and F1 score of genus *Malayemys* classification for each confidence level.

The performance metrics (precision, recall, and F1 score) for the classification of the genus *Malayemys* at varying confidence thresholds provide a comprehensive overview of the model’s effectiveness. Precision measures the accuracy of positive predictions, indicating the model’s ability to minimize false positives. As the confidence threshold increases, the precision typically improves because the model becomes more selective in its predictions, and the best confidence was 0.830. On the other hand, the model’s ability to identify all actual positives, which generally decreases with higher confidence thresholds due to stricter criteria leading to missed true positives was assessed, and the best confidence was less than 0.98. The F1 score, the harmonic mean of precision and recall, balances these two metrics and is particularly useful for understanding overall model performance at each threshold of confidence between 0.49 and 0.98. This analysis is vital for optimizing the model to achieve the best trade-off between missing true positives and incorrectly labeled negatives as positives for application deployment. In summary, as shown in Fig. 5, the peaks with an optimal balance between precision and recall provide crucial insight into the appropriate confidence level at which the model performs best for *Malayemys* classification (between 0.49 and 0.98), aiding in decision-making.

**Fig. 6:**
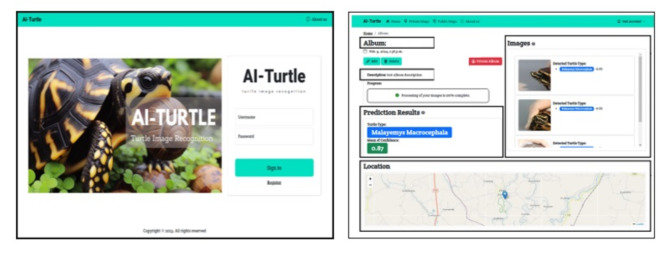
Implementation of automating the transfer of the proposed models to a web-based application (https://ai-turtle.com/**)**.

The practical application of the proposed model has been demonstrated through experiments and their results, as well as through a real-world web application in Fig. 6. This application can perform real-time weighted wAP calculations from the training data of the genus *Malayemys*. Moreover, this application can analyze images from four different single views in a multiview format, yielding high mAP probabilities. The application also displays the probabilities for levels of the mean confidence, with values greater than 0.5. Thus, it is evident that the proposed model operates effectively in real-world scenarios. Recently, this model has been deployed on a website dedicated to our project, allowing scientists to study and share resources more efficiently, thereby enhancing the analysis and discoveries related to the genus *Malayemys*. Moreover, the web application improves location tracking for scientists who discover that a turtle is a type of snail-eating turtle and allows users to switch between public and private mode setting in map visualization.

## Discussion and applications

Turtle species identification plays a crucial role in conservation efforts, with these reptiles serving as effective bioindicators for environmental monitoring. Accurate species identification is essential for detecting native species in ecosystems, forming a critical foundation for environmental analysis and evaluation. Distribution assessment challenges have been encountered for the *Malayemys* genus because of the turtle trade and the difficulty in distinguishing between the three species. We propose a novel ensemble multiview image processing approach for the automated classification of the three *Malayemys* species. This method improves the original YOLOv8 architecture by utilizing a CNN to overcome the limitations of traditional expert-based identification methods. By analyzing *Malayemys* images from multiple angles, this technique captures unique morphological features, addressing challenges such as occlusion and appearance variations. By utilizing a comprehensive dataset, the ensemble multiview strategy significantly improves the YOLOv8 classification accuracy, achieving a mAP of 98% for *Malayemys* species. This performance surpasses that of both nonensemble multiview and single-view strategies. The classification accuracy of this study is higher than other reports such as Gray et al. (2019) developed a convolutional neural network (CNN) specifically for detecting sea turtles in drone imagery, marking a significant advancement in the application of AI to ecological monitoring^24^. The study achieved a notable detection accuracy of 93%, demonstrating the potential of CNNs for automating wildlife surveillance, particularly in marine environments. By leveraging drone imagery, the model successfully overcame traditional challenges of detecting sea turtles over vast areas with minimal human intervention. However, while the results are promising, the study highlights the need for further refinement to improve detection under more complex conditions, such as varying water clarity, lighting, and sea state. Moreover, generalizing the model across different geographic regions and species variations remains a challenge that warrants additional exploration. Badawy and Direkoglu (2019) presented a study on sea turtle detection using the Faster R-CNN model, aiming to support conservation efforts through advanced image recognition techniques^25^. The model achieved an impressive accuracy of 90.2%, demonstrating its efficacy in detecting sea turtles from various imagery sources. The research highlights the potential of Faster R-CNN in accurately identifying turtles even in complex environments, offering a valuable tool for automated wildlife monitoring. Liu et al. (2020) presented the application of convolutional neural networks (CNN) for the classification and recognition of sea turtle images training set, marking a significant advancement in the use of artificial intelligence for wildlife conservation^26^. This research highlights the potential of CNN in addressing the challenges of classifying turtle species and accuracy given 96.4%. However, the performance model across different geographical regions or under diverse environmental factors was not extensively evaluated, limiting its generalizability. Differently, the research primarily focuses on controlled environments and may not fully address challenges posed by real-world conditions such as varying lighting, complex backgrounds, or partial occlusions, which are common in field applications. Baek et al. (2023) proposed other efficient approach for classifying imported turtles in South Korea, utilizing the Single Shot MultiBox Detector (SSD) model in conjunction with various backbone networks^27^. The research findings indicate that the ResNet18 model performed the best, achieving a mean Average Precision (mAP) of 88.1%. Additionally, the model was able to accurately classify turtle species with an average accuracy of 82.8%. Thus, our proposed research demonstrates robustness in varying environments and leverages the capability of multi-view imaging, enhancing decision-making accuracy by avoiding bias toward any single perspective.

The study employs a combination of turtle taxonomy and an AI-assistant methods to provide a novel approach for safeguarding the genetic and species diversity of the genus *Malayemys*. Notably, *M. khoratensis*, endemic to northeastern Thailand and Laos, and *M. macrocephala*, a species of turtle widespread throughout northern, central, eastern, and southern Thailand, and *M. subtrijuga*, a species found in Thailand, Cambodia, Indonesia, Laos, and Vietnam, share characteristics that present challenges in field identification. Therefore, this model will be applied to the accurate determination of these species, enabling the release of these species in an accurate distribution area. The identification application can be transferred to smartphones. The data collected by the program are utilized for several purposes related to population ecology and environmental information, including species distribution, ecological monitoring, habitat health assessment and other issues. We hope that this application decreases the probability of genetic and species diversity loss. Furthermore, the data support researchers in field identification. We believe that this study is a tool for government officers in Thailand to achieve field identification and stop the freshwater turtle trade.

## Conclusion

In this study, we confirm the efficacy of YOLOv8 when integrated with a CNN architecture. This model achieves significant improvements in object detection tasks across various datasets. The method of calculating a weighted mean average precision allows for the effective combination of the strengths of the multiview image strategy in an ensemble, potentially enhancing the overall performance of the object detection model by leveraging the specific capabilities of each model. The YOLOv8 classification of *Malayemys* species is an outstanding accuracy of 98%. This weighted approach provides a more nuanced assessment of the ensemble performance, particularly in complex scenarios where different models may perform calculations when detecting different types of objects. Future work could explore further optimization techniques and extend applications to more complex turtle image recognition tasks, reinforcing the adaptability and robustness of this combined approach in the evolving field of computer science and biology to help scientists accurately characterize turtle species. Moreover, identification accuracy may aid in automatically protecting the species and genetic diversity of the genus *Malayemys* through transfer learning applications.

## Data Availability

The data presented in the study are available upon request from the corresponding author.
